# Supplementary material for: Concatenated Analysis Sheds Light on Early Metazoan Evolution and Fuels a Modern “Urmetazoon” Hypothesis
Source: PLoS Biol. 2009 Jan 27;7(1):e1000020. doi: 10.1371/journal.pbio.1000020 (PMC2631068; doi:10.1371/journal.pbio.1000020)
Supplement: Figure S1 — The shown analysis was done for one of the “plausible” parsimony trees. Other topologies preferred by parsimony analysis gave similar inferences about support. The figure shows whether the partitioned Bremer support values are positive negative or neutral. This figure demonstrates that the nuclear versus mitochondrial partitions all provide similar degrees of support for the various nodes in the tree. Note that over half of the nodes acquire positive support from both partitions (11/21). Most of the negative support in the tree is within the diploblast clade (six out of eight nodes) indicating the instability of the relationships in this clade. Note also that the majority of the negative support comes from mitochondrial partitions further strengthening our contention that the mitochondrial partitions are NOT swamping the nuclear partitions. Nodes at the base of the tree exhibit consistent support from all sources under the shown partitioning scheme. Quite strikingly, nuclear proteins seem to provide the highest positive support of all the characters in the analysis. (70 KB PPT) [file pbio.1000020.sg001.ppt]

## Slide 1
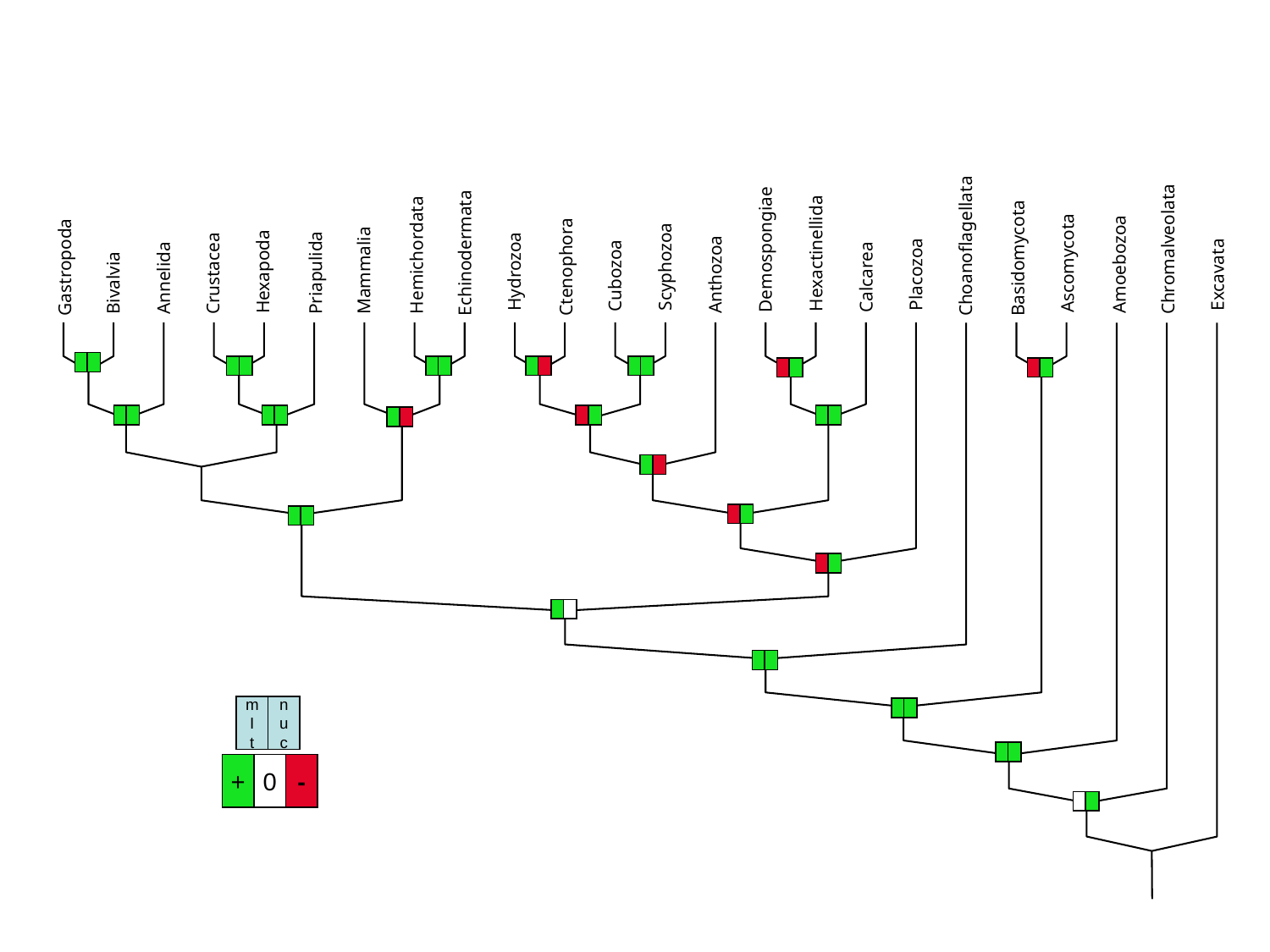

Choanoflagellata
Chromalveolata
Demospongiae
Echinodermata
Hexactinellida
Hemichordata
Basidomycota
Ascomycota
Amoebozoa
Ctenophora
Scyphozoa
Gastropoda
Mammalia
Hydrozoa
Hexapoda
Priapulida
Crustacea
Placozoa
Anthozoa
Excavata
Cubozoa
Calcarea
Annelida
Bivalvia
m
I
t
n
u
c
+
0
-
