# Supplementary figures and images for: Concatenated Analysis Sheds Light on Early Metazoan Evolution and Fuels a Modern “Urmetazoon” Hypothesis
Source: PLoS Biol. 2009 Jan 27;7(1):e1000020. doi: 10.1371/journal.pbio.1000020 (PMC2631068; doi:10.1371/journal.pbio.1000020)

## Slide 1
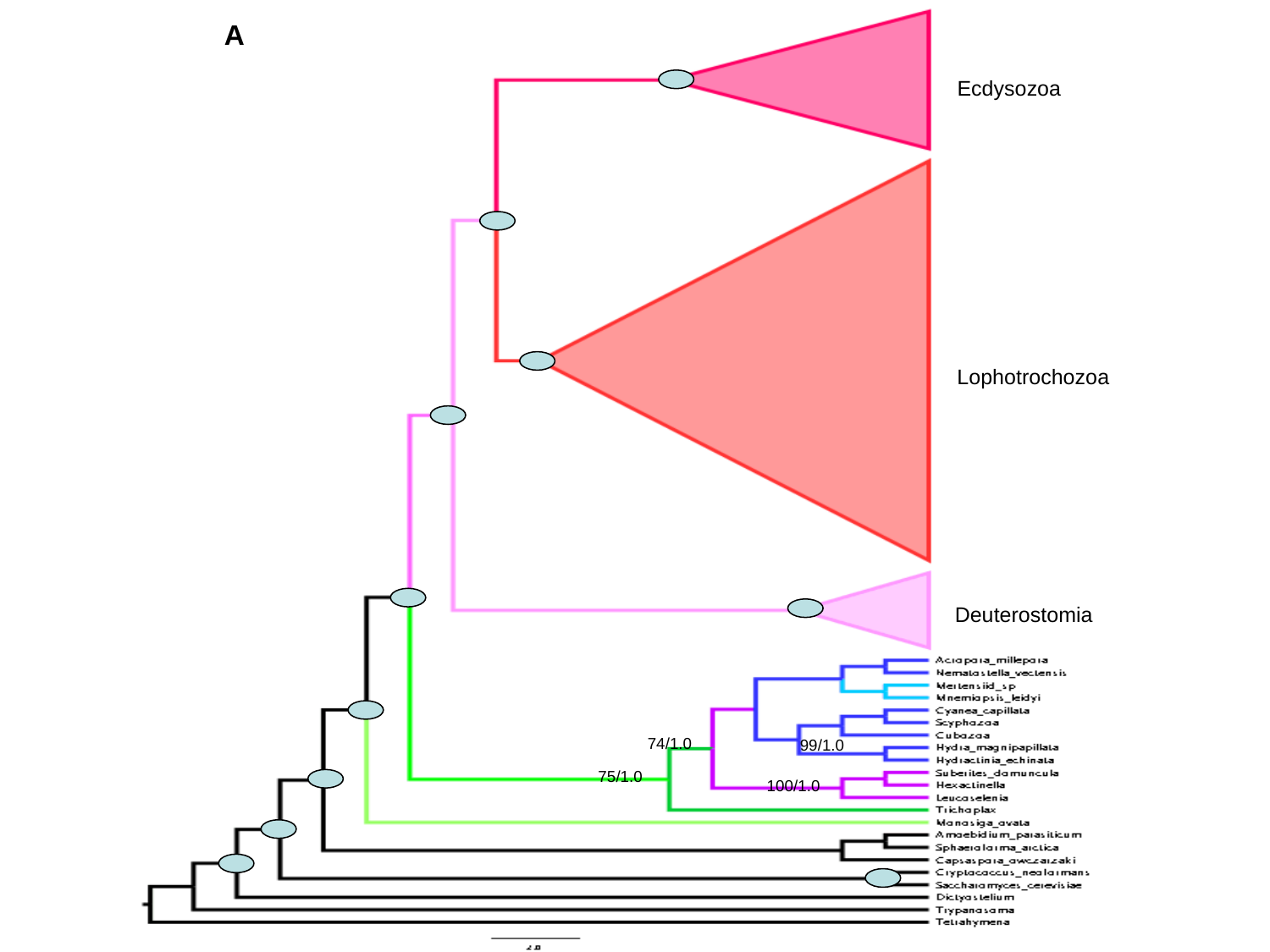

A
Ecdysozoa
Lophotrochozoa
Deuterostomia
74/1.0
99/1.0
75/1.0
100/1.0

## Slide 2
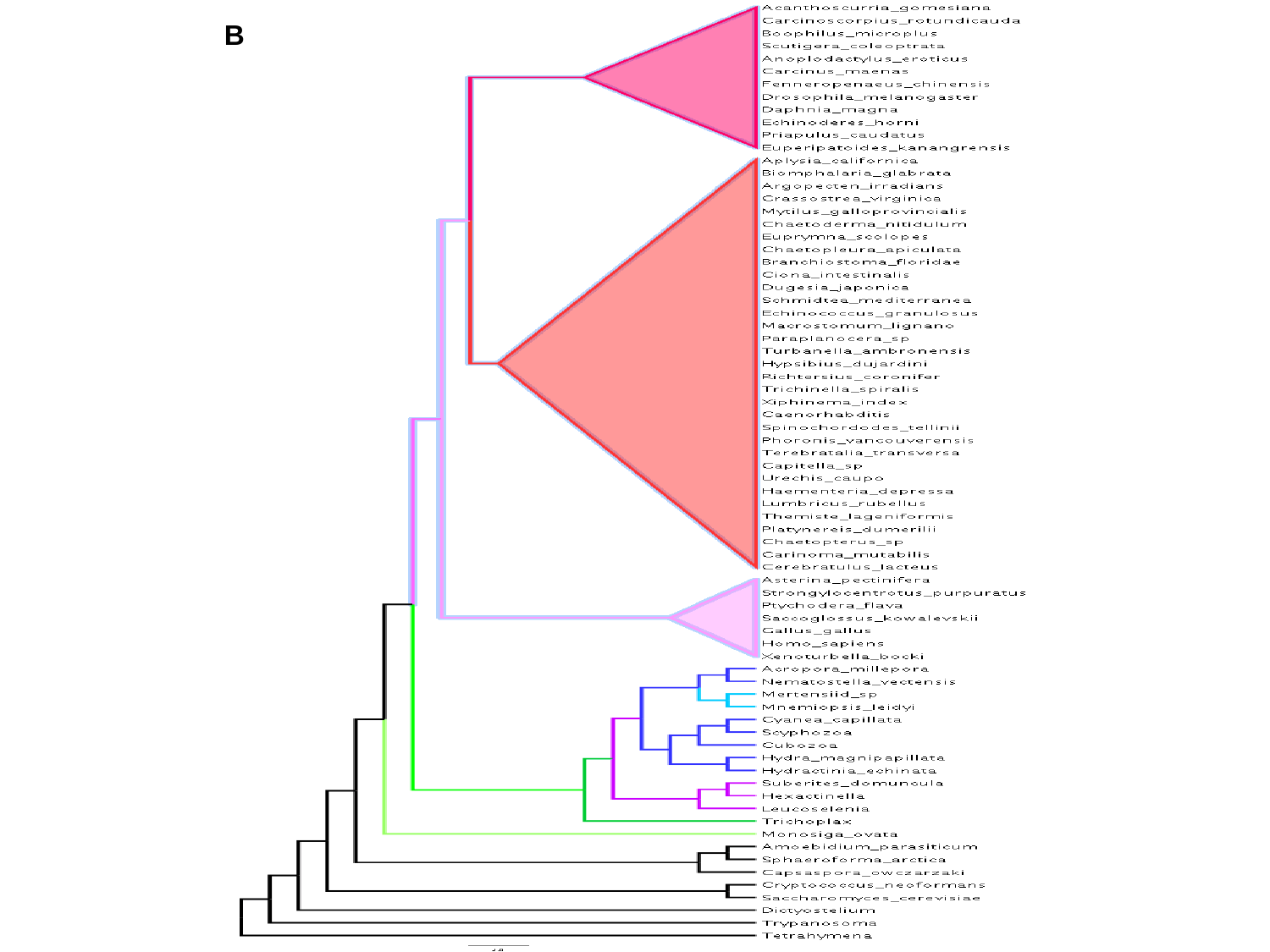

B

Supplement: Figure S2 — The 73 taxa are comprised of the 64 taxa from the Dunn et al. (2008) study [25] plus nine taxa added from the present study. Since the topologies within Lophotrochozoa, Ecdysozoa, and Deuterostomia are not discussed in our study, we have represented these as major monophyletic groups in this figure (A). All included taxa are listed in (B). The blue circles indicate that the support for these nodes are 100% jackknife support for unweighted parsimony analysis and 1.0 posterior Bayesian probability for parsmodel analysis in MrBayes. For four nodes relevant to the present study from this larger analysis, the jackknife values and Bayesian posteriors are listed next to the nodes, respectively. (105 KB PPT) [file pbio.1000020.sg002.ppt]

## Slide 1
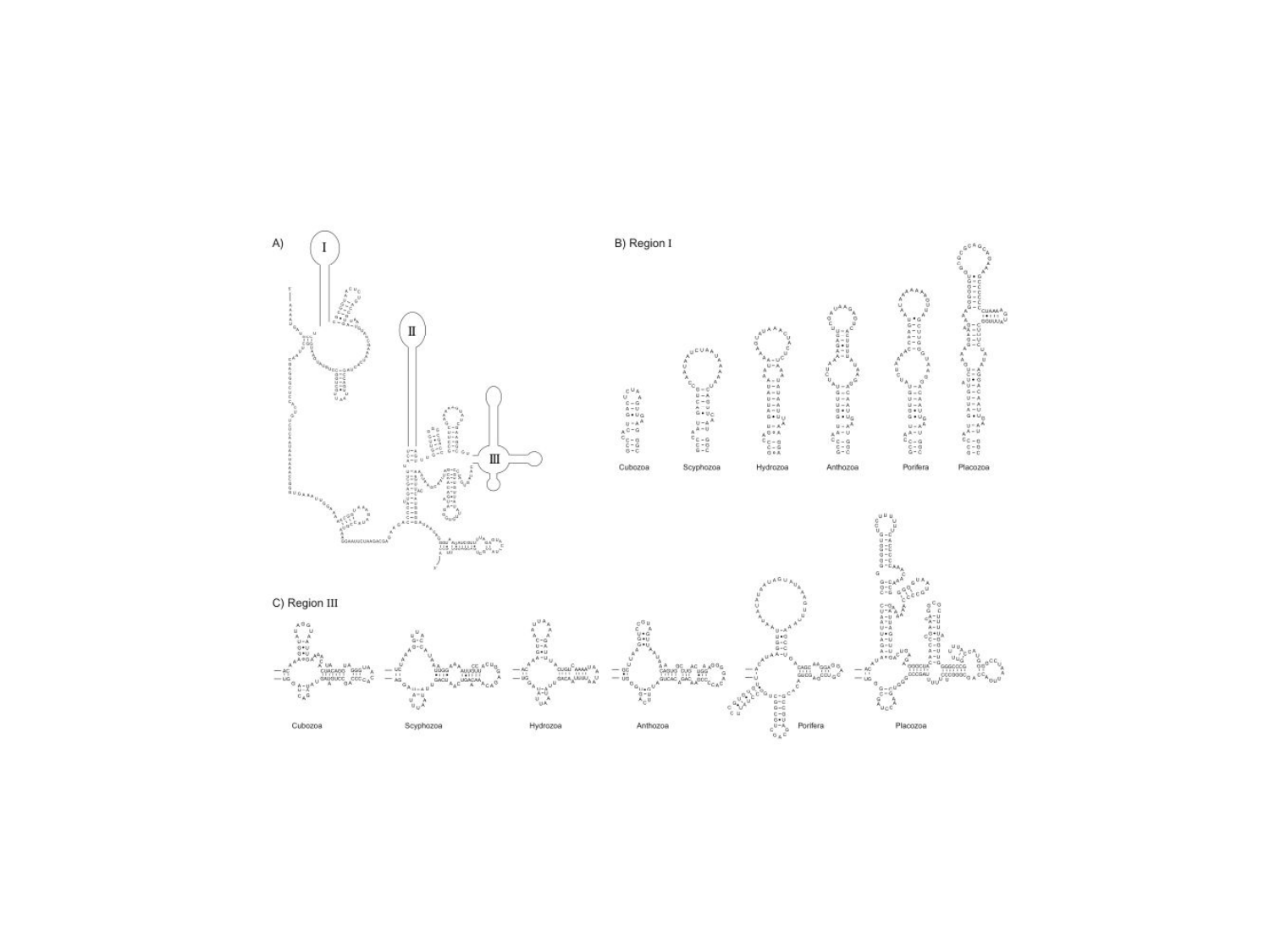

Supplement: Figure S3 — (126 KB PPT) [file pbio.1000020.sg003.ppt]

## Slide 1
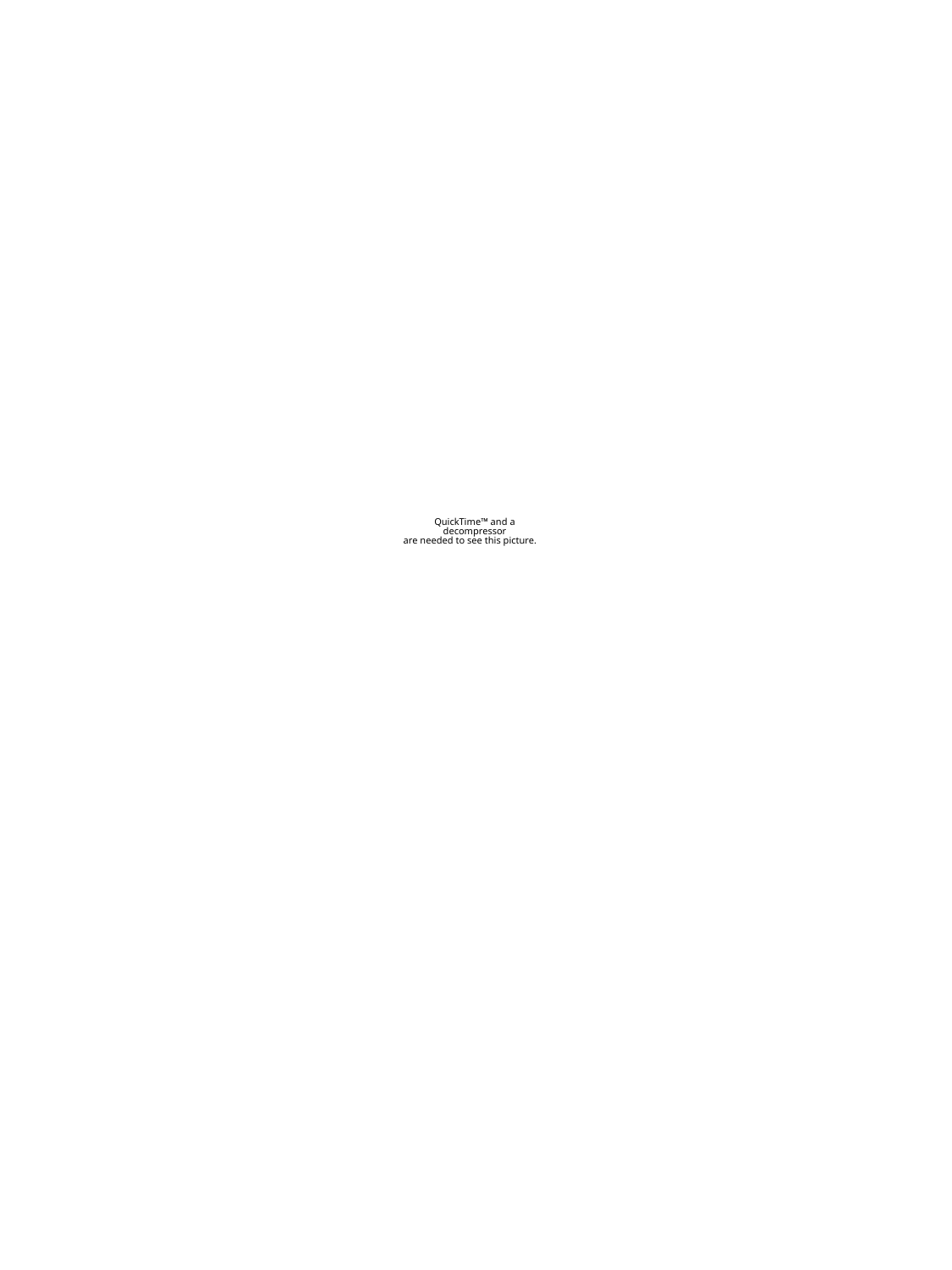

Supplement: Figure S4 — The two Hox-like genes, Cnox-1 and Cnox-3, display differential spatiotemporal expression patterns in the medusa stage. Cnox-1 (A1–A4) is expressed ectodermally in the so-called Nesselring, an area of undifferentiated cells lining the ring canal of medusae (cross section: A3, A4). Cnox-3 expression marks the most ectodermal oral part of the manubrium (B1, B2). Staining is with NBT/X-phosphate (A1, B1) and fluorescein-labeled probes (A2, B2); the scale bar indicates 50 μm. Pictures are reprinted from Jakob and Schierwater (2007) [52]. (2.17 MB PPT) [file pbio.1000020.sg004.ppt]
