# Supplementary material for: Concatenated Analysis Sheds Light on Early Metazoan Evolution and Fuels a Modern “Urmetazoon” Hypothesis
Source: PLoS Biol. 2009 Jan 27;7(1):e1000020. doi: 10.1371/journal.pbio.1000020 (PMC2631068; doi:10.1371/journal.pbio.1000020)
Supplement: Table S3 — (24 KB DOC) [file pbio.1000020.st003.doc]

**Supplementary Figure 3. 16S secondary structure prediction.**

1 2 3 4 5 6 7 8 9 10 11 12 13 14 15 16 17

*Protozoa* 0 0 0 0 0 0 0 0 0 0 0 0 0 0 0 0 0

*Placozoa* 1 1 0 0 0 0 0 0 0 0 0 0 0 0 ? ? 0

*Porifera* 1 2 0 1 1 1 1 1 0 0 0 0 0 0 1 1 1

*Anthozoa* 1 2 1 2 1 1 1 1 1 1 1 1 1 0 1 1 1

*Hydrozoa* 1 2 1 2 1 1 1 1 1 1 1 1 1 1 1 1 1

*Scyphozoa* 1 2 1 2 1 1 1 1 1 1 1 1 1 1 1 1 1

*Cubozoa* 1 2 1 2 1 1 1 1 1 1 1 1 1 1 1 1 1

*Ctenophora* 1 2 2 2 0 ? 1 1 1 1 2 1 1 1 1 1 1

*Bilateria* 1 2 2 2 1 1 1 1 1 1 2 1 1 1 1 1 2

1. SGD: soma-germ-line differentiation (0=exceptionally; 1=always)

2. SOD: intrasomatic differentiation (0=absent, 1=2-5 2=>5 somatic cell types)

3. MUS: contractile cells (0=absent, 1= epithelio-muscle cells, 2= muscle cells)

4. EXC: excitation (conducting) cells (0, 1=in non-specialized cells, 2=nerve cells)

5. TOT: totipotent cell lineages (0, 1)

6. CRD: cell re-differentiation (0, 1)

7. COL: collagen (0, 1)

8. ECM: extracellular matrix (0, 1)

9. BAL: basal lamina (0, 1)

10. DIG: digestive cavity (0, 1)

11. SYM: multicellular symmetry (0=absent, 1=radial, 2=biradial)

12. DBA: defined body axis (0, 1)

13. MOU: mouth and/or anus (0, 1)

14. SEN: sensory organs (0, 1)

15. ECT: ectoderm (0, 1)

16. ENT: entoderm (0, 1)

17. MES: mesogloea (0, 1), mesoderm (2)
